# *Trp53* loss drives the neoplastic transformation of *Pik3ca*^*H1047R*^-induced vascular malformation in a mouse model

**DOI:** 10.1371/journal.pone.0348285

**Published:** 2026-05-04

**Authors:** Miaolu Tang, Jessica Sallavanti, Matthew Lanza, Danielle Covington, Wei Li

**Affiliations:** 1 Division of Hematology and Oncology, Department of Pediatrics, Penn State College of Medicine, Hershey, Pennsylvania, United States of America; 2 Department of Comparative Medicine, Penn State College of Medicine, Hershey, Pennsylvania, United States of America; 3 Department of Cell and Biological Systems, Penn State College of Medicine, Hershey, Pennsylvania, United States of America; 4 Penn State Cancer Institute, Penn State College of Medicine, Hershey, Pennsylvania, United States of America; University of South Carolina, UNITED STATES OF AMERICA

## Abstract

Vascular malformations are anomalies of blood or lymphatic vessels that are frequently associated with activating *PIK3CA* mutations. Although these lesions are generally considered non-neoplastic, rare cases of malignant transformation to angiosarcoma have been reported, and the mechanisms underlying this progression remain unclear. Here, using a conditional mouse model in which *GFAP-Cre*^*ERT2*^ induces *Pik3ca*^*H1047R*^ expression with or without *Trp53* loss, we observed an unexpected cutaneous vascular phenotype rather than intracranial tumor formation. Following tamoxifen induction, blood blister-like lesions developed on the tail, ear, and paw in 86.9% (53/61) of mice harboring at least one *Pik3ca*^*H1047R*^ allele, whereas no lesions were observed in mice lacking the mutant allele (0/13, P < 0.0001). *Trp53* loss did not significantly alter lesion incidence (76.5% vs 70.2%, P = 0.76), indicating that *PIK3CA* activation is sufficient for lesion initiation. Histologically, the lesions consisted of cavernous CD31^+^ vascular channels with frequent thrombosis, most prominently in the dermis, consistent with venous or arteriovenous malformations. Mechanistically, endothelial cells lining the lesions showed little detectable p-AKT signal, whereas adjacent intervascular cells displayed increased p-AKT and focal GFAP expression, suggesting that PI3K activation in non-endothelial intervascular cells contributes to lesion initiation and remodeling. Importantly, *Trp53* deficiency promoted malignant-like progression, with lesions exhibiting endothelial atypia, mitotic activity, intraluminal tufting, and infiltrative growth; 7 of 159 tail lesions showed malignant-like features reminiscent of angiosarcoma. Together, these findings demonstrate that PIK3CA activation initiates highly penetrant vascular malformations, whereas p53 loss promotes their rare neoplastic transformation. This model provides mechanistic and translational insight into how benign *PIK3CA*-mutant vascular malformations may progress toward vascular malignancy and offers a platform for studying biomarkers and therapeutic strategies to prevent this transition.

## Introduction

Vascular malformations are anomalies of blood or lymphatic vessels that arise from errors in vascular development. Unlike vascular tumors, which are characterized by endothelial proliferation, vascular malformations consist of structurally abnormal yet mature vascular channels [1]. Based on the vessel type involved, vascular malformations can be categorized into capillary, venous, lymphatic, arteriovenous, and mixed forms [2]. Because of their persistence and potential for morbidity, vascular malformations pose a considerable clinical challenge.

Molecular studies have revealed recurrent mutations in the PI3K/AKT/mTOR and RAS/RAF/MEK pathways that drive abnormal vascular signaling [3, 4]. In particular, activating mutations in *PIK3CA*, which encodes the catalytic subunit of PI3K, have been identified in both venous and lymphatic malformations [5–10]. Mouse models have become indispensable for studying the pathogenesis and therapeutic interventions of these lesions [11]. Conditional expression of *Pik3ca*^*H1047R*^ in distinct endothelial lineages has successfully recapitulated venous and lymphatic malformations, including spinal lesions with *Sprr2f-Cre* [7], embryonic mesoderm-driven anomalies with *T-Cre*^*ERT2*^ [8], lymphatic malformations with *Prox1-Cre*^*ERT2*^ or *Vegfr3-Cre*^*ERT2*^ [12], and cutaneous venous malformations with *Vegfr1-Cre*^*ERT2*^ [13].

Although vascular malformations are traditionally considered non-neoplastic, rare reports of angiosarcoma and other vascular malignancies arising in regions of chronic lymphedema, long-standing malformations, or previously treated lesions have raised the possibility of malignant transformation [14, 15]. Whether abnormal endothelium within malformations itself can serve as a tumor precursor remains unresolved, and the mechanisms underlying such events are unknown.

In this study, we employed a mouse model in which *Pik3ca*^*H1047R*^ expression was induced by *GFAP-Cre*^*ERT2*^. We found blood blister-like lesions with features of vascular malformation on the skin. Concurrent loss of *Trp53* promoted neoplastic transformation into lesions with angiosarcoma-like features. These findings suggest that while PIK3CA activation initiates abnormal but benign vascular growth, additional mutations, such as p53 inactivation, can drive neoplastic progression.

## Materials and methods

### Tamoxifen-inducible GEMM

The following mice were purchased from the Jackson Laboratory: *GFAP-creT2/ + : B6.Cg-Tg (GFAP-cre/ERT2) 505Fmv/J* (JAX# 012849) [16], *Kras(STOP)/ + Trp53(LoxP/+): B6.129-Kras*^*tm4Tyj*^ *Trp53*^*tm1Brn/*^*J* (JAX# 032435) [17], and *Pik3ca(STOP)/ + : FVB.12*9S6-Gt (ROSA) 26Sortm1 (Pik3ca*H1047R) Egan/J (JAX# 016977) [18]. A series of crosses were performed to generate *GFAP‑CreERT2* mice carrying *Pik3ca*^*H1047R*^ and *Trp53*^*LoxP*^ alleles in all zygosity combinations (heterozygous or homozygous for each allele). To induce genetic mutations, tamoxifen (Cayman Chemical, 13258) was dissolved in corn oil overnight at 37 °C at a concentration of 20 mg/ml. 100 µl of tamoxifen solution was administered to the mice via intraperitoneal injection once daily for five consecutive days at 4–6 weeks of age. All animals were housed in a room under a 12-h light/dark cycle, with free access to a standard rodent diet and water, at ambient temperature (18–23 °C) and humidity (40–60%). Humane endpoints were used for mice developing the blood blister lesions under the skin. The endpoints were set as when the lesions on the tail reach up to 1 cm in length, or before, or if the blisters lead to difficulty in ambulating, drinking, or eating, or the animal fails to groom. Once reaching these endpoints, the mice will be euthanized by CO_2_ inhalation immediately. No mice died before meeting these criteria for euthanasia. 53 mice in the experiments were euthanized by following the endpoint criteria, and the experiment duration was up to 160 days. The mice’s health and behavior were monitored daily by veterinarians and trained investigators. All experiments described in this study were carried out with the approval of the Penn State University Institutional Animal Care and Use Committee and in accordance with its guidelines.

### Genotyping

Tissue collection and digestion: chemically disinfected scissors were used to collect <3 mm of the tip of a toe from each pup (between 5–7 days of age) for genotyping. A different toe was collected from each mouse to allow for mouse identification. Samples were then digested in 100 µL of digestion solution (1 mg/mL proteinase K, 1X genotyping buffer [2x buffer: 67 mM Tris HCl, pH 8.8, 16.6 mM ammonium sulfate, 6.7 mM magnesium chloride, 5 mM β-Mercaptoethanol], 1% Triton X-100) for 3 h at 55 °C at 450 rpm. Proteinase K was inactivated by heating at 95 °C for 10 min, followed by centrifugation at room temperature at 15,000 rpm for 10 min.

Standard PCR: standard PCR for genotype identification of *GFAP‑Cre*^*ERT2*^ and *Trp53*^*LoxP*^ was conducted using PCR ProFlex PCR System. Genomic DNA was added to 1x DreamTaq PCR Master Mix (Thermo scientific, k1082) and 5 µM primers. PCR samples were run on an agarose gel (1X TAE, 2% agarose (Fisher, BP160−500), 0.5 x10^-4^% ethidium bromide (Fisher, BP1302−10)) at 100 volts for 45 min. Bands were exposed using ENDURO™ GDS. The primer sequences used were: *Trp53*^*LoxP*^ (F: GGTTAAACCCAGCTTGACCA, R: GGAGGCAGAGACAGTTGGAG), *GFAP‑Cre*^*ERT2*^ (Transgene F: GCCAGTCTAGCCCACTCCTT, Transgene R: TCCCTGAACATGTCCATCAG, Internal Positive Control: CTAGGCCACAGAATTGAAAGATCT, Internal Positive Control: GTAGGTGGAAATTCTAGCATCATCC). Band sizes: Trp53 (Mutant: 390 bp, Wildtype: 270 bp), *GFAP‑Cre*^*ERT2*^ (Transgene = ~200 bp, Positive Internal Control = 324 bp).

Taqman PCR: to genotype *Pik3ca*^*H1047R*^, genomic DNA was diluted to 5 ng/µL in 0.4 µM primers (Common F: CTGGCTTCTGAGGACCG, Mutant R: CGAAGAGTTTGTCCTCAACCG, Wildtype R: AATCTGTGGGAAGTCTTGTCC), 0.15 µM Taqman probes (Mutant Probe: ACCCTGGACTACTGCGCCC with 5’ VIC and 3’ quencher, Wildtype Probe: TAACCTGGTGTGTGGGCGTTGT with 5’ FAM and 3’ quencher), and 1x TaqMan™ Gene Expression Master Mix (Thermo scientific, 4370048). Taqman PCR was conducted on CFX96 Touch Real-Time PCR Detection System and analyzed using Bio-Rad CFX Manager.

### Histopathological evaluation

Mouse tissue samples were fixed in 4% neutral-buffered formalin for 2 days, then transferred to 70% ethanol. Tail and limb samples were additionally treated with Immunocal Decalcifier (StatLab, 1414–32 QRT) for 5–7 days after fixation. Tail samples consisted of approximately 20% of the total tail length, with sections chosen to include the most affected sites. Tissues were then submitted to Penn State College of Medicine’s Comparative Medicine Histology Core for paraffin embedding and sectioning at 5 μm for slide preparation and routine hematoxylin and eosin (H&E) staining.

The tissues from 12 mice were submitted to a board-certified veterinary pathologist (M.L.) for histopathological evaluation using an OLYMPUS BX51 microscope and an OLYMPUS DP28 microscope camera. Evaluation consisted of descriptive histomorphologic evaluation based on published criteria for histopathological features for diagnosing vascular tumors and vascular malformations [19–23], including the ISSVA (International Society for the Study of Vascular Anomalies), and the International Harmonization of Nomenclature and Diagnostic Criteria for Lesions in Rats and Mice (INHAND Project). As definitive diagnosis of the specific vascular anomaly requires additional testing (e.g., imaging to evaluate patterns of blood flow through the lesions), a conservative approach to diagnosis was employed by classifying the lesions into broad histomorphologic categories (i.e., cavernous, vessel bundles, or irregular) with each category given a narrow list of possible diagnoses based on the histological features of the lesions. A confident diagnosis of malignancy was diagnosed based on the diagnostic criteria established by consensus of the aforementioned research groups, focusing on cellular atypia, mitotic activity, and local tissue invasiveness (e.g., breaking through the basement membrane, or extending from the dermis into the medullary cavity of the subjacent bone). Note that specific criteria for malignancy vary by lesion. The cross-sectional area of lesions was determined using the closed polygon measuring tool in OLYMPUS cellSens Standard 4.2.1 (Build 29742). An approximate percentage of tissue affected was calculated by the sum of the areas of the lesions divided by the total cross-sectional area of the tissue sections on the slide, reported to the nearest 5%. Semiquantitative scoring was performed to assess the severity of secondary changes including the degree of inflammation around the lesions and the degree of bone loss/lysis. Semiquantitative scoring was also used to assess histologic features of the cells within the lesions that are most frequently associated with neoplasia and malignancy (i.e., mitotic activity and cellular atypia). For each of these criteria, the scoring ranged from 0 (unremarkable), 1 (minimal), 2 (mild), 3 (moderate), and 4 (severe). The specific criteria for each of the histopathological features reported in this study are as follows: For inflammation, 0 = no additional inflammatory cells than in normal immune surveillance for that tissue; 1 = minimal inflammation; 2 = mild inflammation; 3 = moderate inflammation; 4 = severe inflammation. For decreased bone, 0 = no bone loss/lysis observed; 1 = minimal bone loss/lysis (small, focal lysis <1% of the total area of the affected bone); 2 = mild bone loss/lysis (<10% of the total area of the affected bone); 3 = moderate bone loss/lysis (10–20% of the total area of the affected bone); 4 = severe bone loss/lysis (>20% of the total area of the affected bone). For increased Mitotic Activity, 0 = no mitotic Figs observed within the lesion; 1 = minimal mitotic activity (1 mitotic Fig within the lesion and/or within up to 10 consecutive 400x microscopic fields of a lesion); 2 = mild mitotic activity (2–3 mitotic Figs within up to 10 consecutive 400x microscopic fields of a lesion but <3 mitotic Figs per single 400x microscopic field); 3 = moderate mitotic activity (<10 mitotic Figs within up to 10 consecutive 400x microscopic fields of a lesion but <5 mitotic Figs per single 400x microscopic field); 4 = severe mitotic activity (>10 mitotic Figs within up to 10 consecutive 400x microscopic fields of a lesion or >5 mitotic Figs per single 400x microscopic field). For cellular atypia, 0 = no cellular atypia; 1 = minimal cellular atypia (rare karyomegaly and/or variation in cell size); 2 = mild cellular atypia (multiple but <10% of cells within a lesion exhibit significant karyomegaly, variation in cell size, and/or increased nuclear:cytoplasmic (N:C) ratio); 3 = moderate cellular atypia (10–30% of cells within a lesion exhibit significant karyomegaly, variation in cell size, and/or increased N:C ratio; bizarre mitotic Figs may be present but are very rare); 4 = severe cellular atypia (>30% of cells are poorly differentiated with frequent karyomegaly, generalized anisocytosis, and/or generalized increased N:C ratio; multiple bizarre mitotic Figs are present).

### Chromogenic immunodetection

Additional unstained paraffin-embedded 5-µm sections were deparaffinized and rehydrated in successive baths of xylene and ethanol (100%, 95%, 70%, and 50%), followed by heat-based (95 °C) epitope retrieval in 10 mM sodium citrate buffer (pH = 6.0). Slides were rinsed with dH_2_O and rehydrated with wash buffer (0.1% Triton X-100 in DPBS). Next, slides were incubated with a peroxidase suppressor (Thermo Scientific, 35000) for 15–30 min and washed with wash buffer for 5 min. The slides were then incubated in 5% normal goat serum in wash buffer for 30 min, followed by avidin and biotin blocking (Vector Laboratories, SP-2001) for 15 min each. Staining was carried out using primary antibodies diluted (1:100–1:200) in incubation buffer (2.5% BSA, 0.05% Triton X-100 in DPBS) at 4 °C overnight. The next day, after three washes with wash buffer for 5 min each, the biotinylated goat anti-rabbit IgG antibody (Vector Laboratories, BP-9100–50) was applied to the slides for 30 min. After another three washes, slides were incubated with a mixture of reagents A and B (Vector Laboratories, PK-6200) for 30 min. Targeted protein expression is then visualized through incubation in ImmPACT DAB reagent (Vector Laboratories, SK-4105) for 2–10 min, followed by counterstaining with hematoxylin solution to visualize the cell nucleus. The slides were then mounted using VectaMount Express Mounting Medium (Vector Laboratories, H-5700–60) after dehydration with 99% isopropanol. Antibody used: CD31 (Cell Signaling Technology, 77699), GFAP (Cell Signaling Technology, 12389), αSMA (Cell Signaling Technology, 19245), Ki-67 (Cell Signaling Technology, 9129), p53 (Santa Cruz Biotechnology, sc-126), ERG (Cell Signaling Technology, 97249), PROX1 (Novus Biologicals, NBP1–30045), p-AKT (Signaling Technology, 4060).

### Immunofluorescent staining

The staining protocol was described in [24]. Briefly, paraffin-embedded tissue was sequentially immersed in xylene and ethanol (100%, 95%, 70%, and 50%), followed by a rinse with dH_2_O for deparaffinization and rehydration. After heating at a sub-boiling temperature in a 10 mM sodium citrate buffer (pH = 6.0) for antigen retrieval, samples were rinsed with dH_2_O and rehydrated using a wash buffer (0.1% Triton X-100 in DPBS). The samples were incubated with blocking buffer (2.5% BSA, 0.05% Triton X-100 in PBS) at room temperature for 1 h, followed by incubating with primary antibodies diluted in blocking buffer (1:50–1:100) overnight at 4°C. After washing three times with wash buffer (0.1% Triton X-100 in DPBS, 10 min each), samples were incubated with a secondary antibody diluted in the blocking buffer (1:100–1:200) for 1 h at room temperature. After washing three times with wash buffer (0.1% Triton X-100 in DPBS, 10 min each), samples were incubated with DAPI for 1 min and mounted in ProLong^TM^ Gold Antifade Mountant (Invitrogen, P10144). Images were acquired with a Leica SP8 confocal microscope using a 63x oil-immersion lens with a 1 µm optical section. Images shown were from 3 sections projected. Antibody used: CD31 (Santa Cruz Biotechnology, sc-376764) and a-SMA (Cell Signaling Technology, 19245). For triple staining, the antibodies used: αSMA (Cell Signaling Technology, 60839), CD31 (Cell Signaling Technology, 66477), Ki-67 (Signaling Technology, 12075).

### Statistical analysis

All statistical calculations and plotting were performed using GraphPad Prism (v11). Statistical significance was determined as indicated in the figure legends or the text. Unless otherwise indicated, all center values shown are mean values, and all error bars represent the standard errors of the mean (SEM). No sample size estimation was performed. No data were excluded from the analyses. All animals were maintained in the same environment and handled by the same procedure.

## Results

### *GFAP-Cre*^*^ERT2^*^-induced *Pik3ca*^*H1047R*^ expression causes blood blisters on the skin of multiple body parts

**Fig 1:**
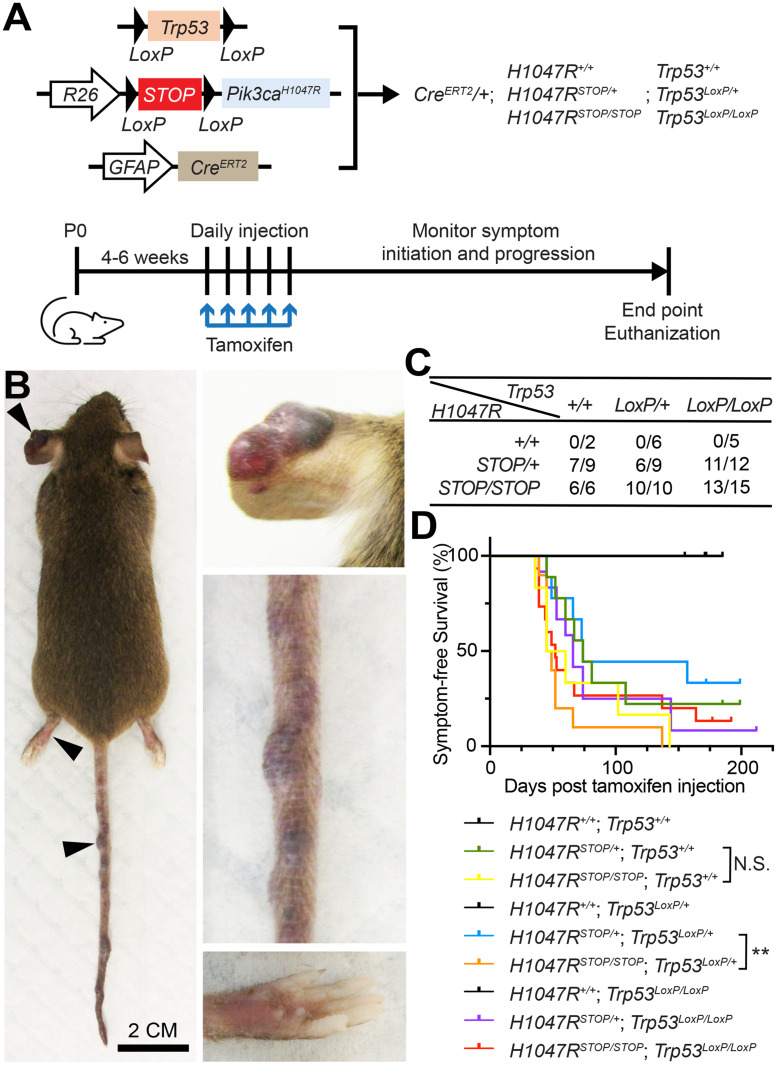
*GFAP-Cre^ERT2^*-induced *Pik3ca^H1047R^* expression causes blood blisters on the skin of multiple body parts. **(A)** Schematics of the experiment plan and the Cre-loxP system used to introduce the expression of *Pik3ca*^*H1047R*^ and the loss of *Trp53*. **(B)** Representative images of the blood blisters on multiple body parts of a mouse with *GFAP-Cre*^*ERT2*^; *Pik3ca*^*H1047R/+*^; *Trp53* ^*LoxP/LoxP*^ genotype. **(C)** The number of mice that developed blood blister lesions out of the total number of mice in each genotype. All mice have *GFAP-Cre*^*ERT2*^. A total of 74 mice from 13 or more litters were scored in the study. **(D)** Blood blistering symptom-free survival curve of mice with different genotypes after tamoxifen injection. **N.**S., p > 0.05; **, p < 0.01, Log-rank test.

*PIK3CA* and *TP53* are among the most frequently mutated genes in glioblastoma [25]. To examine whether targeted introduction of *PIK3CA* and *TP53* mutations into astrocytes can induce glioblastoma, we employed GEMM lines containing *Pik3ca*^*H1047R*^ [18] and *Trp53*^*LoxP*^ [17] mutations, respectively. In addition, mice containing the *GFAP-Cre*^*ERT2*^ transgene [16] were used to achieve conditional expression of Cre (Fig 1A). Following tamoxifen injection at 4–6 weeks of age, we did not observe the development of intracranial tumors in any mouse strain during the follow-up period (up to 30 weeks). Unexpectedly, many of the mice developed blood-filled blister-like lesions on the skin of multiple body parts, including the tail, paw, and ear (Fig 1B). When comparing mice with various combinations of mutation alleles, we found that none (0 out of 13) of the mice without the *Pik3ca*^*H1047R*^ allele developed blood blisters during the 30-week follow-up period (Fig 1C). In contrast, 86.9% (53 out of 61) of mice with at least one *Pik3ca*^*H1047R*^ allele developed the blood blisters. This observation suggests that *Pik3ca*^*H1047R*^ expression causes the lesion (P < 0.0001, Fisher’s exact test). In addition, 76.5% (13 out of 17) of the mice without the *Trp53*^*LoxP*^ allele and 70.2% (40 out of 57) of the mice with the *Trp53*^*LoxP*^ allele showed the lesion, suggesting *Trp53* loss is not associated with the lesion (P = 0.76, Fisher’s exact test) (Fig 1C). Among those mice with at least one *Pik3ca*^*H1047R*^ allele, 82.6% (19 out of 23) of female and 89.5% (34 out of 38) of male mice developed the blood blisters, indicating no (P = 0.46, Fisher’s exact test) sex difference in the lesion incidence. Mice with homozygous *Pik3ca*^*H1047R*^ alleles (*GFAP-Cre*^*ERT2*^; *Pik3ca*^*H1047R/H1047R*^) developed such lesions slightly earlier than those heterozygous in *Pik3ca*^*H1047R*^ (*Cre*^*ERT2*^; *Pik3ca*^*H1047R/+*^), although the difference is not statistically significant (P = 0.27, Log-rank test). Interestingly, this difference was significantly increased (P = 0.0067, Log-rank test) when one *Trp53* allele was lost (Fig 1D). However, loss of both *Trp53* alleles did not increase the difference (P = 0.58, Log-rank test), suggesting that lesion development is sensitive to the dosage of *Pik3ca*^*H1047R*^ under hypoactive *Trp53*.

### Blood blister lesions are primarily found in the dermis

**Fig 2:**
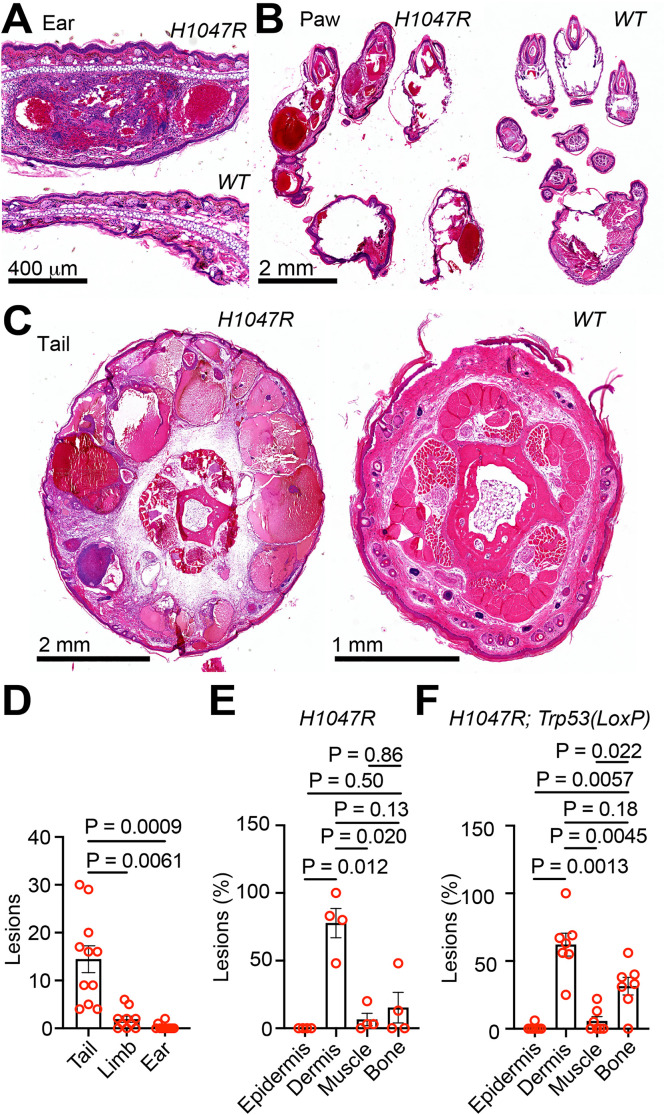
Blood blister lesions are primarily found in the dermis. **(A)** H&E staining of the ears of a *GFAP-Cre*^*ERT2*^; *Pik3ca*^*H1047R/+*^; *Trp53*^*+/+*^ mouse (top) and a B6 wild-type (WT) lesion-free mouse (bottom). **(B)** H&E staining of the paws of a *GFAP-Cre*^*ERT2*^; *Pik3ca*^*H1047R/+*^; *Trp53*^*+/+*^ mouse (left) and a B6 wild-type (WT) lesion-free mouse (right). **(C)** H&E staining of the tail of a *GFAP-Cre*^*ERT2*^; *Pik3ca*^*H1047R/+*^; *Trp53*^*+/+*^ mouse (left) and a B6 wild-type (WT) lesion-free mouse (right). **(D)** The number of lesions found in each indicated organ during the histological examination of the mice shown in S1 Table. n_tail_ = 11; n_limb_ = 10; n_ear_ = 11. (E) and (F) the percentage of lesions distributed in the indicated tissues of the tail during the histological examination of the mice shown in S1 Table. In **(E)**, *GFAP-Cre*^*ERT2*^; *Pik3ca*^*H1047R/+*^ *or Pik3ca*^*H1047R/H1047R*^; *Trp53*^*+/+*^ mice (n = 4) were counted. In **(F)**, *GFAP-Cre*^*ERT2*^; *Pik3ca*^*H1047R/+*^ *or Pik3ca*^*H1047R/H1047R*^; *Trp53*^*LoxP/+*^ or *Trp53*^*LoxP/LoxP*^ mice (n = 7) were counted. One-Way ANOVA, followed by Dunnett’s multiple comparisons test.

In further analysis of the blood blisters, we found that the lesions on the ear, paw, and tail are histologically most consistent with vascular malformations, consisting of cavernous dilations of abnormal blood vessels that often exhibit thrombosis (Figs 2A-C). The distribution of lesions varied by anatomic location (S1 Table), with the tails exhibiting the highest lesion counts (Fig 2D). In the tail, the lesions were found in the epidermis, dermis, muscle, and bone. Counting the number of lesions in different tail tissues of the mice only with the *Pik3ca*^*H1047R*^ allele, we found that most of the lesions were in the dermis, while there was no difference in lesion counts among the epidermis, bone, and muscle (Fig 2E). In mice carrying both the *Pik3ca*^*H1047R*^ and *Trp53*^*LoxP*^ alleles, lesions were also mainly found in the dermis; however, lesion frequency in the bone was higher than in the epidermis and muscles (Fig 2F).

To examine whether the vascular malformation also occurs in other organs, we systemically assessed 3 *GFAP-Cre*^*ERT2*^; *Pik3ca*^*H1047R/H1047R*^; *Trp53*^*LoxP/LoxP*^ mice. H&E histology assessment of liver, kidneys, heart, lungs, spleen, pancreas, and brain did not find any vascular anomalies or other vascular lesions in these mice. The gastrointestinal and genitourinary tracts of the two mice examined also showed no vascular lesions (S1 Table). These results indicated that the lesions are primarily a skin-limited phenotype and are not associated with systemic vascular disease.

### Vascular malformation underlies the blood blisters

Because the lesions appeared more frequently and were more visible on the tails, we focused the subsequent analysis on tail lesions. Histopathological evaluation was performed in 12 mice (S1 Table), revealing that the blood blisters consist of a heterogeneous set of lesions, including abnormal vascular structures, which overall comprise three general histomorphologic patterns.

**Fig 3:**
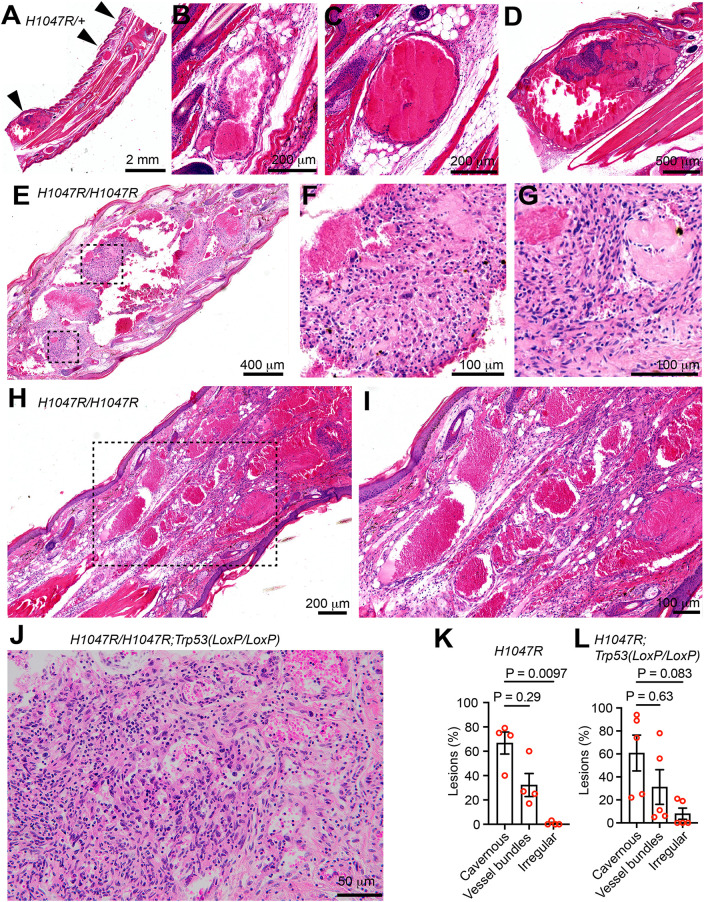
Vascular malformation underlies the blood blisters. **(A)** H&E staining of the tail of a *GFAP-Cre*^*ERT2*^; *Pik3ca*^*H1047R/+*^; *Trp53*^*+/+*^ mouse. **(B-D)** Each lesion of different sizes indicated in (A) was magnified and shown. **(E)** H&E staining of the tail of a *GFAP-Cre*^*ERT2*^; *Pik3ca*^*H1047R/H1047R*^; *Trp53*^*+/+*^ mouse. **(F and G)** The areas outlined in (E) were zoomed in and shown. **(H)** H&E staining of the tail of a *GFAP-Cre*^*ERT2*^; *Pik3ca*^*H1047R/H1047R*^; *Trp53*^*+/+*^ mouse. **(I)** The area outlined in (H) was zoomed in and shown. **(J)** H&E staining of the tail of a *GFAP-Cre*^*ERT2*^; *Pik3ca*^*H1047R/H1047R*^; *Trp53*^*LoxP/LoxP*^ mouse. (K) and (L) the percentage of each indicated lesion type found in the tail dermis during the histological examination of the mice shown in S1 Table. In **(K)**, *GFAP-Cre*^*ERT2*^; *Pik3ca*^*H1047R/+*^ *or Pik3ca*^*H1047R/H1047R*^; *Trp53*^*+/+*^ mice (n = 4) were counted. In **(L)**, *GFAP-Cre*^*ERT2*^; *Pik3ca*^*H1047R/+*^ *or Pik3ca*^*H1047R/H1047R*^; *Trp53*^*LoxP/LoxP*^ mice (n = 5) were counted. One-Way ANOVA, followed by Tukey’s multiple comparisons test.

The first pattern is comprised of cavernous blood-filled spaces surrounded by a thin layer of endothelial cells and occasionally subdivided into smaller spaces by thin septa (Figs 3A-D). Morphologically, these lesions resemble intraosseous hemangiomas or thin-walled vascular malformations. These lesions were observed in the tails of 10 of 12 mice, for a total of 104 lesions (ranging from 2 to 23 per affected mouse), representing approximately 70% of the total cross-sectional area of tail lesions. 4 of 104 lesions in the tail demonstrated partial thrombosis and central papilliferous nodules composed of spindle-shaped cells with large nuclei and increased mitotic activity, surrounded by bland endothelial cells, resembling Masson’s tumors, also referred to as pseudoangiosarcomas or intravascular papillary endothelial hyperplasia (Figs 3E-G). Cavernous lesions were also observed in the distal limbs of 4 of 11 mice, for a total of 6 lesions (ranging from 1 to 2 per affected mouse), representing approximately 45% of the total cross-sectional area of limb lesions.

The second type consists of bundles of abnormal, blood-filled vascular structures with relatively well-differentiated cells and some degree of recognizable arterial, venous, and/or capillary architecture (Figs 3H-I). These lesions most closely resemble vascular malformations, including arteriovenous, venous, or capillary malformations or arteriovenous fistulas. These lesions were observed in the tails of 11 of 12 mice, for a total of 46 lesions (ranging from 1 to 9 per affected mouse), representing approximately 30% of the total cross-sectional area of tail lesions. These lesions were also observed in the distal limbs of 5 of 11 mice, for a total of 13 lesions (ranging from 1 to 4 per affected mouse), representing approximately 55% of the total cross-sectional area of limb lesions (S1 Table).

The third histomorphologic pattern consists of densely packed streams of spindle-shaped cells that form irregular vascular channels, lacking the recognizable organization of normal blood vessels (Fig 3J). Cells within these irregular lesions often exhibit cellular atypia (i.e., anisocytosis, anisokaryosis, increased nuclear:cytoplasmic ratio) and increased mitotic activity. These lesions were observed in the tails of 3 of 12 mice, for a total of 8 lesions (ranging from 1 to 4 per affected mouse), representing approximately 5% of the total cross-sectional area of tail lesions. This lesion type was not seen within the limbs of these mice (S1 Table). These lesions histologically resemble solid subtypes of vascular anomalies.

Among the three histomorphologic patterns (lesions), ~ 60% and 30% belong to the type 1 (cavernous) lesions and type 2 (vessel bundles) lesions, respectively, although the percentage difference of these two lesions is not significant (Figs 3K-L). Type 3 (irregular) lesions are rare (0.75%) in mice with the *Pik3ca*^*H1047R*^ allele alone (Fig 3K). However, 8% of type 3 lesions are observed in mice carrying *Trp53*^*LoxP/LoxP*^ and the *Pik3ca*^*H1047R*^ allele, suggesting that *Trp53* loss promotes the development of type 3 lesions. Overall, lesions from *GFAP-Cre*^*ERT2*^; *Pik3ca*^*H1047R*^ mice are mostly consistent with arteriovenous or venous malformations.

### A combination of *Pik3ca*^*H1047R*^ mutation and *Trp53* loss drives the neoplastic transformation of vascular malformation

**Fig 4:**
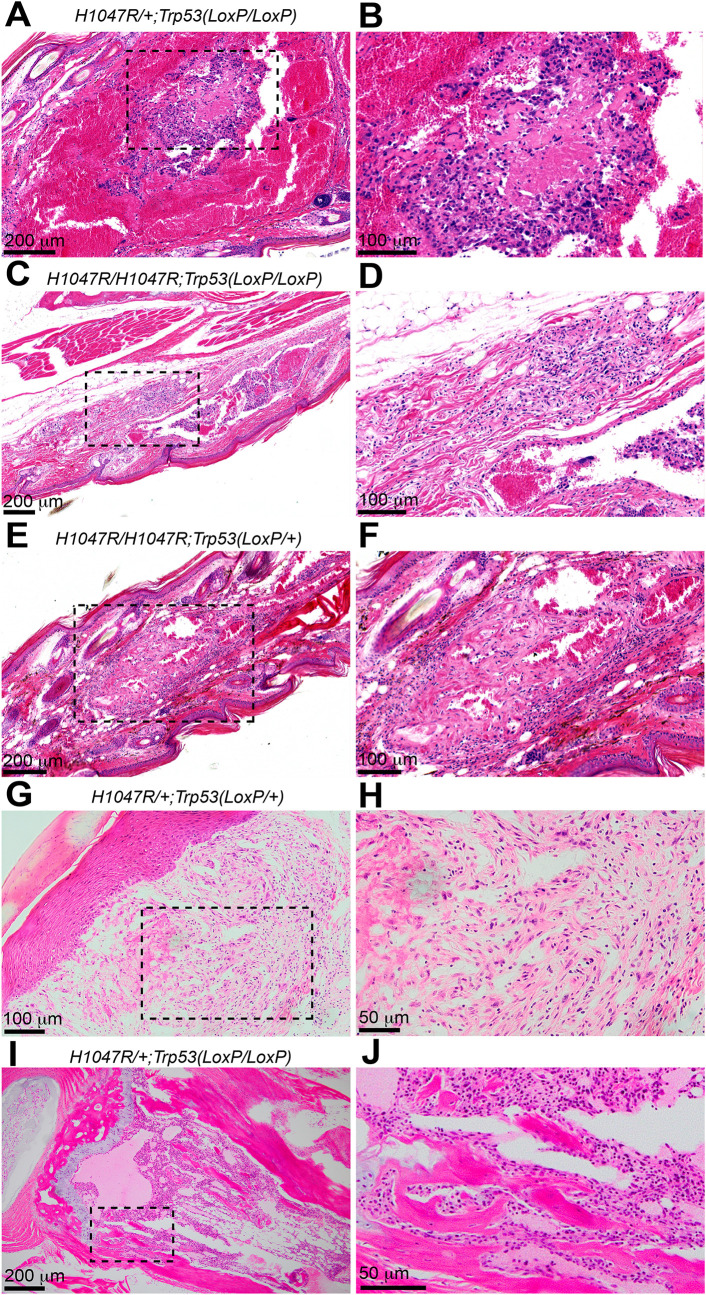
A combination of *the Pik3ca*^*H1047R*^ mutation and *Trp53* loss drives malignant transformation in vascular malformations. (A) and **(B)** H&E staining of the tail sections of *GFAP-Cre*^*ERT2*^; *Pik3ca*^*H1047R/+*^; *Trp53* ^*LoxP/LoxP*^ mice. (C) and **(D)** H&E staining of the tail sections of *GFAP-Cre*^*ERT2*^; *Pik3ca*^*H1047R/H1047R*^; *Trp53* ^*LoxP/LoxP*^ mice. (E) and **(F)** H&E staining of the tail sections of *GFAP-Cre*^*ERT2*^; *Pik3ca*^*H1047R/H1047R*^; *Trp53*^*LoxP/+*^ mice. (G) and **(H)** H&E staining of the tail sections of *GFAP-Cre*^*ERT2*^; *Pik3ca*^*H1047R/+*^; *Trp53*^*LoxP/+*^ mice. (I) and **(J)** H&E staining of the bone sections of *GFAP-Cre*^*ERT2*^; *Pik3ca*^*H1047R/+*^; *Trp53* ^*LoxP/LoxP*^ mice. The areas outlined in (A, C, E, G, I) were zoomed in and shown in (B, D, F, H, **J)**, respectively.

Although loss of *Trp53* did not increase the frequency of the blood blisters triggered by *Pik3ca*^*H1047R*^ mutation (Fig 1C, P = 1, Fisher’s exact test), we found that lesions in 3 of 5 mice with *Pik3ca*^*H1047R*^ mutation and *Trp53*^*LoxP/LoxP*^ exhibited features more commonly seen with malignancy. In comparison, lesions in 0 of 4 *Pik3ca*^*H1047R*^-containing mice without the *Trp53*^*LoxP*^ allele show malignant features (S1 Table). This suggests a trend (P = 0.17, Fisher’s exact test) that *Trp53* loss promotes malignancy in the presence of *Pik3ca*^*H1047R*^ mutation. These cavernous lesions, showing malignant features, often have multiple blood-filled spaces with large thrombi, containing varying degrees of cellular organization, and are associated with infiltrates of proliferating fibroblasts and inflammation at the centers of the spaces (Figs 4A and 4B). The cavernous lesions in the dermis have multiple, central, irregular proliferations of densely packed smooth muscle and/or endothelial cells that extend into the lumen (Figs 4C and 4D). These cells exhibit increased cellular atypia, a nuclear-to-cytoplasmic ratio, and mitotic activity, and occasionally wrap around collagen, a feature commonly observed in angiosarcoma (Figs 4E and 4F). One of the cavernous lesions in the dermis is infiltrative and extends into the underlying muscle, suggesting malignancy (Figs 4G and 4H). Lesions in the bones also involve extensive lytic destruction of both trabecular and cortical bone sections with effacement by these atypical cells (Figs 4I and 4J). These observations suggest that *Trp53* loss may drive neoplastic transformation in vascular malformations harboring *Pik3ca*^*H1047R*^*.* Notably, lesions with malignant features, including moderate to severe cellular atypia, mild to moderate increased mitotic activity, and local invasiveness, were observed in 7 of 159 tail lesions from three mice, suggesting that the risk of neoplastic transformation remains low.

### Amplified endothelial and smooth muscle cells are associated with vascular malformation

**Fig 5:**
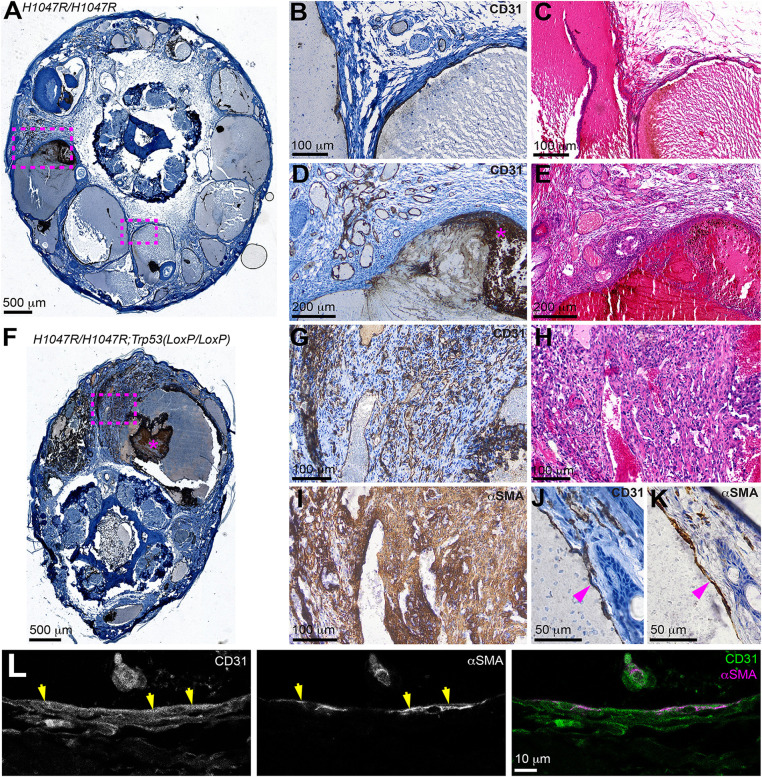
Endothelial cells and smooth muscle cells are involved in the malignant transformation. **(A)** A representative image of chromogenic immunodetection of CD31 in the tail of a *GFAP-Cre*^*ERT2*^; *Pik3ca*^*H1047R/H1047R*^; *Trp53*^*+/+*^ mouse. (B) and **(D)** The areas outlined in (A) were magnified and shown. (C) and **(E)** H&E staining of the adjacent sections from the same tissue shown in (B) and **(D)**, respectively. **(F)** A representative image of chromogenic immunodetection of CD31 in the tail of a *GFAP-Cre*^*ERT2*^; *Pik3ca*^*H1047R/H1047R*^; *Trp53* ^*LoxP/LoxP*^ mouse. **(G)** The area outlined in (F) was zoomed in and shown. (H) and **(I)** H&E (H) and αSMA (I) staining of the adjacent sections from the same tissue shown in **(G)**. (J) and **(K)** Chromogenic immunodetection of CD31 (J) and αSMA (K) in the adjacent sections from the same tissue in the tail of a *GFAP-Cre*^*ERT2*^; *Pik3ca*^*H1047R/H1047R*^; *Trp53* ^*LoxP/LoxP*^ mouse. **(L)** Immunofluorescent staining of CD31 and αSMA in the tail section of a *GFAP-Cre*^*ERT2*^; *Pik3ca*^*H1047R/H1047R*^; *Trp53* ^*LoxP/LoxP*^ mouse.

The above histological analysis suggests that endothelial distortion is associated with the vascular malformation, which is subsequently followed by neoplastic transformation with endothelial cell amplification. Using the endothelial cell marker CD31, we found that the distorted large vascular channels in *GFAP-Cre*^*ERT2*^; *Pik3ca*^*H1047R*^ mice were lined by CD31^+^ endothelial cells (Figs 5A-C). Some of the vascular channels were filled with CD31^+^ cells (Figs 5D and 5E, asterisk). Channels of varying caliber were also lined by CD31^+^ cells (Figs 5D and 5E). When examining lesions in *GFAP-Cre*^*ERT2*^; *Pik3ca*^*H1047R*^; *Trp53*^*LoxP/LoxP*^ mice, we observed more frequent appearance of the clustered CD31^+^ cells in the lumen of the distorted vascular channels (Fig 5F, asterisk). Unlike *GFAP-Cre*^*ERT2*^; *Pik3ca*^*H1047R*^ mice, the lesions in *GFAP-Cre*^*ERT2*^; *Pik3ca*^*H1047R*^; *Trp53* ^*LoxP/LoxP*^ mice were filled with massive distorted microchannels lined by CD31^+^ cells. Many of these CD31^+^ cells do not appear to form vascular channels. These CD31^+^ cells are embedded in dense hypercellular CD31^-^ tissues (Figs 5G and 5H).

Vascular smooth muscle cells are major components of blood vessels. To examine whether vascular smooth muscle cells are involved in neoplastic transformation, we stained for α-smooth muscle actin (αSMA), a commonly used marker of vascular smooth muscle cells. In the lesions of *GFAP-Cre*^*ERT2*^; *Pik3ca*^*H1047R*^; *Trp53*^*LoxP/LoxP*^ mice, cells were largely positive to αSMA staining (Fig 5I), suggesting that amplification of smooth muscle cells contributes to the neoplastic transformation. Interestingly, the CD31^+^ endothelial cells lining distorted vascular channels appeared to be also labeled by αSMA in an adjacent tissue section (Figs 5J and 5K, arrowheads). Using co-immunofluorescence staining, we confirmed that lesion cells can be co-stained by both CD31 and αSMA (Fig 5L, arrowheads).

**Fig 6:**
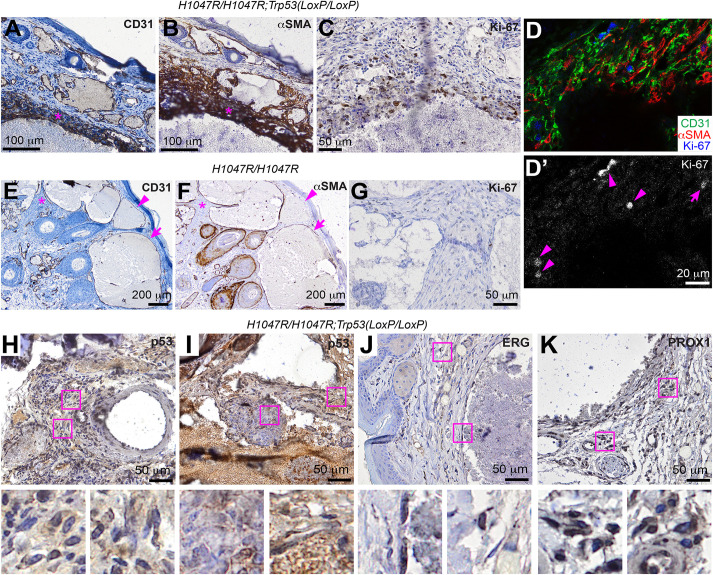
Proliferation of endothelial cells and smooth muscle cells are associated with the malignant transformation. **(A)**-(C) Chromogenic immunodetection of CD31 **(A)**, αSMA **(B)**, and Ki-67 (C) in the adjacent sections from the same tissue in the tail of a *GFAP-Cre*^*ERT2*^; *Pik3ca*^*H1047R/H1047R*^; *Trp53*^*LoxP/LoxP*^ mouse. (D) and (D’) Immunofluorescent staining of CD31, αSMA, and Ki-67 in a section from the tail of a *GFAP-Cre*^*ERT2*^; *Pik3ca*^*H1047R/H1047R*^; *Trp53*^*LoxP/LoxP*^ mouse. **(D)** Merged image; (D’) Ki-67 channel. **(E)**-(G) Chromogenic immunodetection of CD31 **(E)**, αSMA **(F)**, and Ki-67 (G) in the adjacent sections from the same tissue in the tail of a *GFAP-Cre*^*ERT2*^; *Pik3ca*^*H1047R/H1047R*^; *Trp53*^*+/+*^ mouse. **(H)**-(K) Chromogenic immunodetection of p53 (H) and **(I)**, ERG **(J)**, and PROX1 (K) in tail sections from *GFAP-Cre*^*ERT2*^; *Pik3ca*^*H1047R/H1047R*^; *Trp53*^*LoxP/LoxP*^ mice. The outlined areas in each panel were enlarged and shown at the bottom.

In areas lining more severely distorted channels, CD31^+^ and αSMA^+^ cells become multilayered (Figs 6A and 6B, asterisks), indicating amplification by these cells. To examine whether these cells exhibit hyperproliferation, we used the Ki-67 cell proliferation marker. Those multilayered cells showed increased Ki-67 signal, although most of the lesion tissues, including those benign lesions, were negative for Ki-67 (Fig 6C). To examine the identity of these Ki-67^+^ cells, we used co-immunofluorescence staining. The results showed that Ki-67^+^ cells exist in both CD31^+^ and αSMA^+^ cell populations (Figs 6D and 6D’, arrowheads and arrow, respectively), suggesting that the proliferation of both cell populations contributes to the amplified tissues.

To examine whether cells lining the lesion in *GFAP-Cre*^*ERT2*^; *Pik3ca*^*H1047R*^ mice also express αSMA, we performed similar αSMA staining. Most of the intervascular tissues and certain CD31^+^ endothelial cells lining the distorted vascular channels are negative for αSMA (Figs 6E and 6F, asterisks and arrowheads, respectively). However, most of those CD31^+^ endothelial cells lining the distorted vascular channels were associated with αSMA^+^ cells (Figs 6E and 6F, arrows). Similar to *GFAP-Cre*^*ERT2*^; *Pik3ca*^*H1047R*^; *Trp53*^*LoxP/LoxP*^ mice, these monolayered CD31^+^ and αSMA^+^ cells were not stained by Ki-67 (Fig 6G). These results suggest that increased amplification of endothelial cells and smooth muscle cells is limited to the lesions of *GFAP-Cre*^*ERT2*^; *Pik3ca*^*H1047R*^; *Trp53*^*LoxP/LoxP*^ mice.

To further characterize the lesions of *GFAP-Cre*^*ERT2*^; *Pik3ca*^*H1047R*^; *Trp53*^*LoxP/LoxP*^ mice, we examined the expression of p53. Cells in the intervascular tissues of the lesion and the intraluminal tufts were largely negative for the p53 staining (Figs 6H and 6I, respectively), suggesting that the *Trp53* gene is lost in these cells. When staining for ETS-related gene (ERG), a transcription factor and marker of endothelial cell differentiation [26], ERG^+^ cells were found lining the distorted vasculature and interspersing in the lesions (Fig 6J). Prospero Homeobox 1 (PROX1) is a transcription factor driving lymphatic endothelial differentiation [27]. We found that a large proportion of intervascular cells were stained for PROX1 (Fig 6K), suggesting that lymphatic endothelial cells are involved in lesion formation.

### Intervascular cells express GFAP and show increased PI3K activity

**Fig 7:**
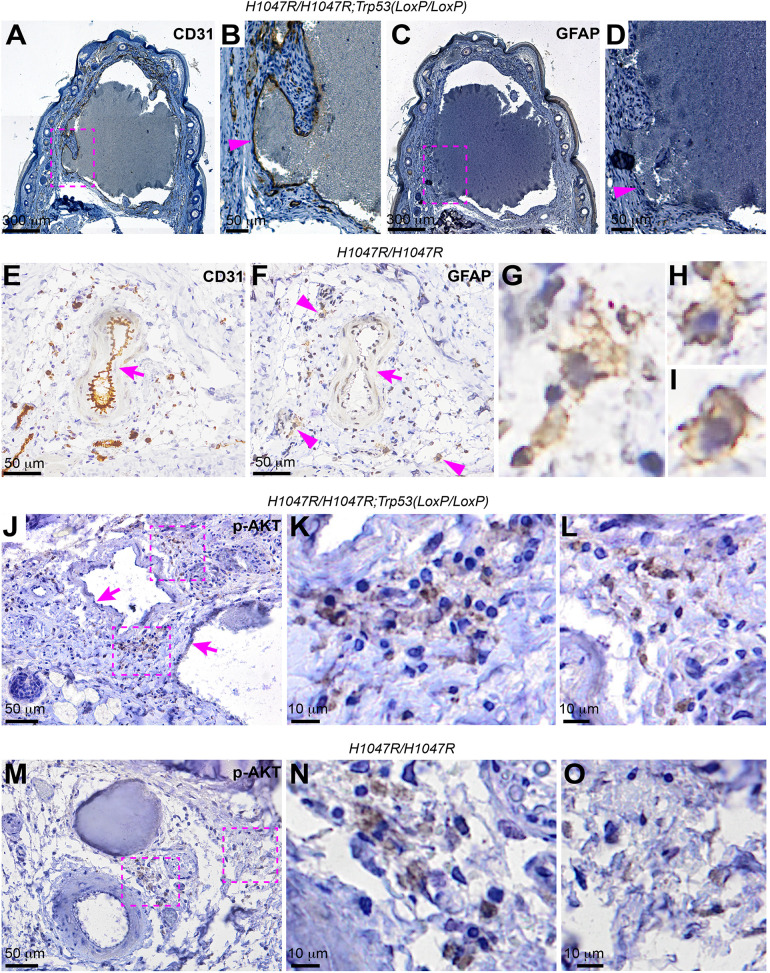
Intervascular cells express GFAP and show increased PI3K activity. (A) and **(B)** Representative images of chromogenic immunodetection of CD31 in the tail of a *GFAP-Cre*^*ERT2*^; *Pik3ca*^*H1047R/H1047R*^; *Trp53*^*LoxP/LoxP*^ mouse. (C) and **(D)** Chromogenic immunodetection of GFAP in the adjacent sections of (A) and **(B)**. The areas outlined in (A) and (C) were magnified and shown in (B) and **(D)**, respectively. (E) and **(F)** Representative images of chromogenic immunodetection of CD31 (E) and GFAP (F) in the tail of a *GFAP-Cre*^*ERT2*^; *Pik3ca*^*H1047R/H1047R*^; *Trp53*^*+/+*^ mouse. Areas pointed by the arrowheads in (F) were individually magnified and shown in **(G)**-(I). **(J)** Representative image of chromogenic immunodetection of p-AKT (S473) in the tail section of a *GFAP-Cre*^*ERT2*^; *Pik3ca*^*H1047R/H1047R*^; *Trp53* ^*LoxP/LoxP*^ mouse. (K) and **(L)** The areas outlined in (J) were magnified and shown in (K) and **(L)**. **(M)** Representative image of chromogenic immunodetection of p-AKT (S473) in the tail section of a *GFAP-Cre*^*ERT2*^; *Pik3ca*^*H1047R/H1047R*^; *Trp53*^*+/+*^ mouse. (N) and **(O)** The areas outlined in (M) were magnified and shown in (N) and **(O)**.

It was reported that GFAP-expressing progenitor cells contribute to the development of vascular smooth muscle cells and certain vascular endothelial cells [28]. This suggests that *GFAP-Cre*^*ERT2*^ may be expressed in these progenitors, in which *Pik3ca*^*H1047R*^ activation *and* *Trp53* loss were induced. When examining whether the distorted endothelial cells express GFAP, we found that CD31^+^ endothelial cells lining the distorted vascular channel were not labeled by a GFAP antibody (Figs 7A-D). To examine if GFAP could be expressed in endothelial cells at an earlier developmental stage when the tamoxifen was administered to induce the Cre-mediated recombination, we stained the mice’s tails at the age of 3 weeks. Although CD31 can label both major and capillary vessels, these CD31^+^ cells were not reactive to the GFAP antibody (Figs 7E and 7F, arrows). However, certain sporadic cells in the dermis were labeled by the GFAP antibody (Figs 7F-I, arrowheads). To examine whether the lesions are associated with increased PI3K activity, we stained them with an antibody against phosphorylated AKT, p-ATK (S473). Endothelial cells lining the lesions were negative for p-AKT (S473) staining (Fig 7J, arrows); however, certain intervascular cells around the blood blister lesion showed increased p-AKT (S473) signals (Fig 7J-L). Similarly, increased p-AKT (S473) signals were observed in certain intervascular cells around the lesions in *GFAP-Cre*^*ERT2*^; *Pik3ca*^*H1047R*^ mice (Figs 7M-O). These results suggest that certain cells other than endothelial cells per se are responsible for initiating and fueling the lesion in response to increased PI3K activity.

## Discussion

Our study identifies an unexpected consequence of *Pik3ca*^*H1047R*^ expression induced by *GFAP-Cre*^*ERT2*^ in certain dermal intervascular cells, which drives the formation of vascular malformations rather than intracranial gliomas. The results also indicate that loss of *Trp53* modifies this outcome, not by increasing lesion incidence, but by promoting the malignant-like conversion of otherwise benign malformations into angiosarcoma-like vascular tumors. These results underscore the principle that the phenotypic consequence of oncogenic mutations is shaped not only by lineage context but also by cooperating genetic alterations.

Consistent with prior studies linking *PIK3CA* mutations to venous and lymphatic malformations, we observed blood blister-like dermal lesions characterized by cavernous, dilated vascular channels with frequent thrombosis. Histologically, these lesions closely resemble arteriovenous or venous malformations, although additional diagnostics with analysis of blood flow would provide greater differentiation and allow for a more specific diagnosis. The high penetrance of lesions in *Pik3ca*^*H1047R*^ mice, regardless of *Trp53* status, establishes PIK3CA activation as the initiating driver. Interestingly, lesion onset was accelerated by *Trp53* haploinsufficiency, suggesting a gene-dosage effect and an interaction with the p53 pathway in modulating disease progression. Although *Trp53* loss alone did not increase lesion incidence, our findings indicate that it conferred malignant potential to otherwise benign vascular malformations. In *Pik3ca*^*H1047R*^; *Trp53*-deficient mice, vascular malformations displayed histologic features of malignancy, including endothelial atypia, mitotic activity, intraluminal tufting, and infiltrative growth into surrounding tissues. In some cases, lesions recapitulated hallmarks of angiosarcoma, such as pericollagenous cell wrapping and high-grade endothelial proliferation. Notably, the relatively infrequent (7 out of 159 tail lesions) malignant-like lesions suggest that neoplastic transformation is a low-probability event superimposed on a background of highly penetrant malformations. This is consistent with the rare cases of malignant transformation to angiosarcoma from vascular malformations observed in the clinic settings. Nevertheless, these results indicate that while PIK3CA activation initiates non-neoplastic vascular malformations, additional loss of tumor suppressors can reprogram these lesions toward neoplasia. This supports the broader hypothesis that vascular malformations, though typically stable, may serve as a substrate for malignant transformation under conditions of acquired genetic “second hits.”

The lesions described in this paper are also reminiscent of human diseases characterized by widespread vascular malformations. Many of the cavernous lesions histologically resemble telangiectasia, such as in hereditary hemorrhagic telangiectasia (HHT), also known as Osler-Weber-Rendu disease. However, these mice did not exhibit the visceral vascular abnormalities as is often seen in this disease. The thin-walled cavernous lesions effacing the medullary cavity of the bone resemble the vascular lesions seen within the bones of patients with Gorham-Stout disease, a rare disease characterized by proliferation of vascular/lymphovascular channels within the bone, which lead to progressive osteolysis [29]. The vertebrae are common locations for this disease, similar to the involvement of the tail vertebrae in these mice. Furthermore, PI3K pathways have been shown to have increased activity in Gorham-Stout disease [30, 31], which suggests that there may be a shared pathogenesis in the development of lesions in this rare disease and in our mouse model. Excessive osteoclast activation is believed to be essential to the pathogenesis of Gorham-Stout disease, and PI3K has been shown to regulate osteoclast activation by increasing the proliferation of osteoclast progenitor cells within the bone marrow [32,33]. KRAS mutations and activation of the RAS/MAPK pathway have been linked to Gorham-Stout disease and vascular anomalies [34,35]. Considering that PI3K is an effector of RAS [36], it is plausible that PI3K activation may account for the osteoclast found in RAS-related Gorham-Stout disease.

Our immunohistochemical data confirmed that the abnormal vascular channels were composed of CD31^+^ endothelial cells. However, these cells lack GFAP expression. The *GFAP-Cre*^*ERT2*^ transgenic mouse line that we used was characterized to drive reporter gene expression in the central nervous system [16]. However, it has been reported that the *GFAP-Cre*^*ERT2*^ line exhibits low specificity for targeting astrocytes [37]. Whether this line shows the Cre activity in peripheral tissues remains unclear. Prior work using constitutively expressed *GFAP-cre* demonstrated robust Cre activity in non-neuronal tissues, including progenitor cells that give rise to vascular smooth muscle and endothelial cells in major arteries [28]**.** Although reporter genes used to trace *GFAP-Cre* activity can be detected in vascular smooth muscle cells and endothelial cells, GFAP expression was not detectable in either cell type. The prior study suggests that Cre-mediated recombination occurred in vascular progenitors, rather than mature endothelial cells [28]. The results of our study have not traced back to the original cells that show *GFAP-Cre*^*ERT2*^ activity and respond to the PIK3CA signal. Therefore, we cannot distinguish whether the distorted cells possess these genetic changes or are indirectly responding to other affected peripheral glial cells that may express *GFAP-Cre*^*ERT2*^. Interestingly, certain intervascular cells showed GFAP expression (Figs 7F-I) and increased p-AKT signals (Figs 7J-L), suggesting that these cells may produce signals that induce vascular malformations. This notion is consistent with the prior report that PI3K activation in vascular connective tissues, such as vascular pericytes, perimysial fibroblasts, and Schwann cell precursors, can induce vascular anomalies [38]. Further studies that incorporate genetically traceable reporters may help identify the cell of origin of the vascular malformations induced in this system.

A limitation of this study is that the cell of origin responsible for lesion initiation has not been defined. Although *GFAP-Cre*^*ERT2*^-driven *Pik3ca*^*H1047R*^ expression induced vascular malformations and *Trp53* loss promoted malignant-like progression, the recombined cell population in peripheral tissues remains incompletely defined. In addition, our classification of the lesions relied primarily on histopathology and immunostaining, without functional vascular imaging or lineage-tracing approaches that would allow more precise distinction among venous, arteriovenous, and lymphatic components and establish whether the proliferating atypical cells arise directly from mutant endothelial/smooth muscle lineages or from neighboring PI3K-activated intervascular cells. The relatively small number of mice and the low frequency of malignant-like lesions also limit the ability to quantify the penetrance and kinetics of neoplastic transformation. Finally, because this phenotype emerged in a *GFAP-Cre*^*ERT2*^-based system rather than a canonical endothelial-restricted model, caution is warranted in extrapolating these findings directly to human vascular malformations. Nonetheless, the results have implications for understanding the rare malignant transformation of vascular malformations reported in clinical settings. While most malformations remain benign, secondary genetic insults, such as *p53* loss*,* may promote progression to vascular malignancy. This model offers a platform to dissect the molecular steps underlying benign-to-malignant transitions and provides a basis for testing targeted therapies, such as PI3K pathway inhibitors [39, 40], in combination with strategies that restore p53 function or mitigate downstream proliferative signaling.

## Supporting information

S1 Table Lesion characterization in mice with different genotypes. (XLSX)
